# Item-Position Binding Capacity Limits and Word Limits in Working Memory: A Reanalysis of Oberauer (2019)

**DOI:** 10.5334/joc.193

**Published:** 2022-01-06

**Authors:** Nelson Cowan

**Affiliations:** 1 Department of Psychological Sciences, University of Missouri, McAlester Hall, Columbia, MO 65211, USA

**Keywords:** Working memory, Short-term memory, Visual word processing

## Abstract

Oberauer (2019) suggested that the working memory capacity in word lists only limits the binding of words to serial positions, with no limit for the words themselves. I advocate a word item limit as a broad kind of binding of each word to the current trial. I propose that the word capacity limit can be observed in Oberauer’s data when binding of a word to the trial is crucial (Experiment 2, words drawn from a small pool and often repeated across trials), though probably much less so when this kind of binding is unimportant (Experiment 1, words drawn from a large pool and rarely repeated across trials). In Oberauer’s recognition procedure for lists of 2, 4, 6, or 8 words, the number of words in the response set was varied, including both words from the list (1, 2, 4, 6, or 8 of them) and words that were not from the list (0, 1, 2, or 4 of them). There was also a serial recall procedure. In a re-analysis of the data from Experiment 2, an overlooked item capacity limit was found that affected the distribution of erroneous responses. Specifically, when the correct answer was unknown to the participant (which happened more at longer list lengths), proportionally fewer words from the list were selected as responses; selection of non-list words increased. It is an important theoretical refinement of Oberauer’s position to include evidence of a word item capacity limit when the item-to-trial binding is crucial, as in his Experiment 2.

An important but unresolved issue regarding human working memory is the nature of capacity limits (e.g., Adam et al., 2017; Cowan, 2001; Cowan et al., 2012, 2020; Hardman et al., 2017; Ma et al., 2014; Nosofsky & Donkin, 2016; Oberauer et al., 2018; Oberauer & Lin, 2017; Pratte et al., 2017; Schurgin et al., 2020). Recently, Oberauer (2019) made an interesting contribution to this debate in experiments on word list recall and recognition. The conclusion was that capacity is primarily limited not by the number of list items but by the number of associations or bindings between each word and its serial position in the list. In the present note, I re-examine this issue and suggest that Oberauer’s conclusion is not fully in keeping with his evidence. Specifically, there is a set of binding limits that includes not only item-to-serial position binding as Oberauer proposed, but also a broader, item-to-trial binding that limited the number of words remembered from a list in Oberauer’s Experiment 2.

There also must be binding of each word to the experiment as a whole, in both experiments of Oberauer (2019), but there is insufficient evidence to claim that this kind of binding, too, is capacity-limited. Experiment 1 is based on a large pool of words rarely repeated from trial to trial, whereas Experiment 2 is based on a small pool of words often repeated from trial to trial. The latter situation necessitates a kind of item-to-trial binding that I find to have a capacity limit that Oberauer did not detect.

I will review the nature of Oberauer’s procedure and the aspects of the results leading him to deemphasize a role for item capacity limits. I will also consider aspects of the results that seem inconsistent with the conclusion. Last, I will propose an alternative theoretical conception to try to account for all of the results. Abbreviations used in this treatment are shown in ***Table 1***.

**Table 1:** Abbreviations of Variable Names.


*pCorr*	Proportion correct

*pL*	Proportion of error trials in which the selection is made from the list items only

*pEFL*	Proportion of errors from list

*#L*	Number of response choices from the list

*#N*	Number of response choices not from the list

*pLRG*	Proportion of the erroneous list responses that come from random guesses

*k*	Number of items in working memory (with or without their serial positions)

*L*	List length

*R*	Number of incorrect choices available that come from the list


## Oberauer (2019) Procedure and Conclusions

**Figure 1:**
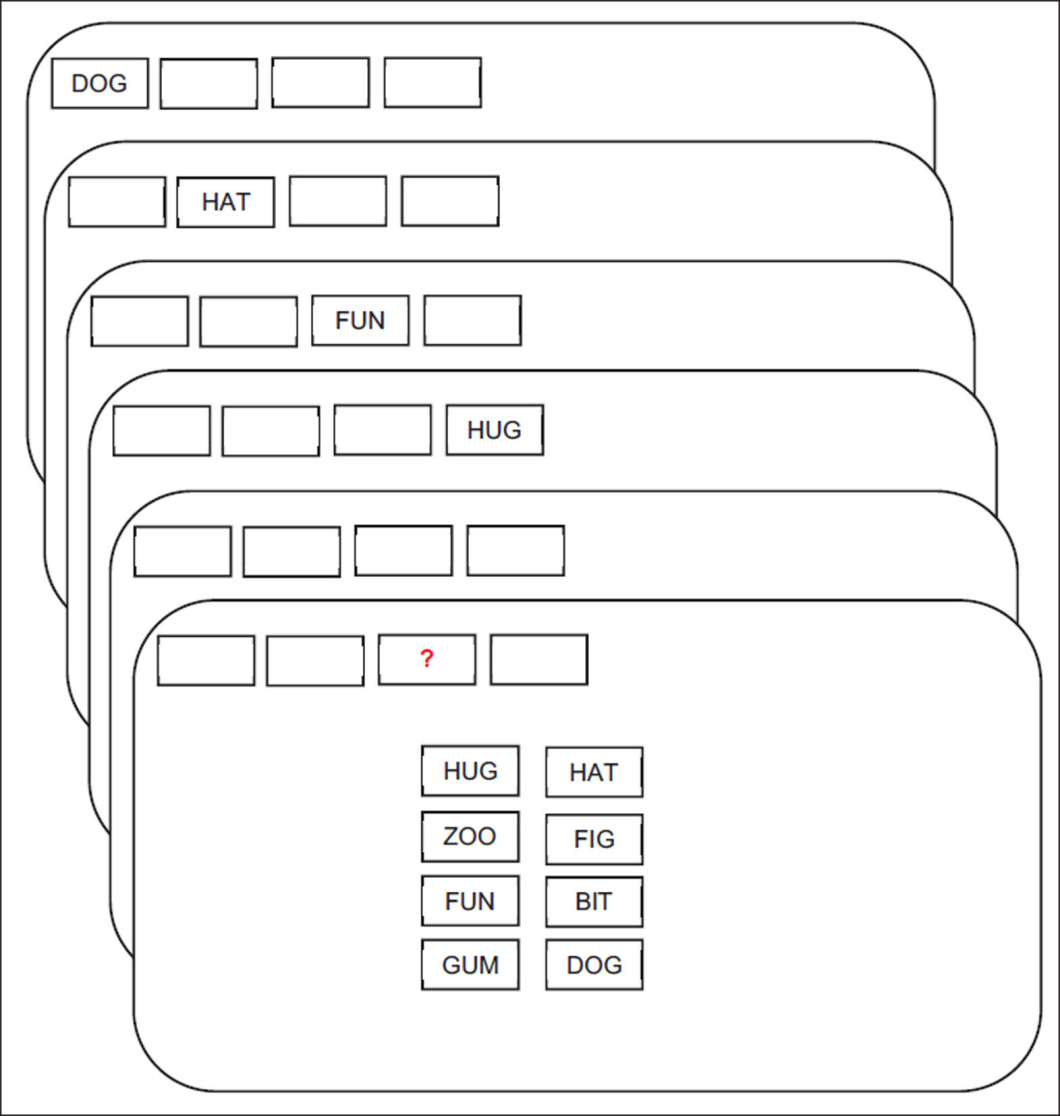
A trial with a list length of 4 words and a response choice set including 4 words from the list and 4 words from outside of the set. List Lengths 2, 4, 6, and 8 were used. When possible, 1, 2, 4, 6, or 8 items from the list appeared in the response set along with 0, 1, 2, or 4 words that were not in the list. There were also trials in which the response was serial recall. Figure reproduced from Oberauer (2019, Figure 1).

Oberauer (2019) carried out two experiments of immediate memory for printed lists in which, after the list, a serial position was cued. The participant was to select a word out of several choices as being the word presented in that serial position (***Figure 1***). Response choices for recognition included the words from the list at the right position, words from other list positions, or words that had not been included in the list at all. There were 2, 4, 6, or 8 words per list and two aspects of the response display were varied: the number of response choices from the list and the number of response choices that were words not from the list. If these are termed *#L* and *#N*, the choice set configuration *#L&#N* included 1&1, 2&0, 2&2, 4&0, 4&2, 4&4, 6&0, 6&2, 6&4, 8&0, 8&2, and 8&4. The trial types were limited by the list length so that, for example, for 4-word lengths there were no 6&_ or 8&_ trials. There was also a serial recall condition. The conclusion that capacity limits came from item-serial position binding limits was based on several findings: the fact that the proportion correct did not much change with the addition of lures that were not in the list, the observation that extra-list errors did not increase much with set size (list length), and the result of measurement models converging on near-zero estimates of the set-size effect on item memory.

As Oberauer (2019, p. 2) explained, “In the large-pool experiment, item memory required discriminating the words seen in the present trial from new words never seen in the entire experiment. This could be accomplished by an episodic-memory record of the words experienced in the experimental setting, without distinguishing between the current trial and previous trials. In the small-pool experiment, item memory required discriminating the words in the current list from the words seen in other recent trials.”

If the item-to-trial binding of Experiment 2 were not problematic, then the two experiments should have produced quite similar responding. However, Oberauer acknowledges that they were dissimilar. For example, he indicated (p. 10): “This is not to say that memory for items was perfect – in the small-pool experiment it clearly was not. Yet, whatever limits item memory does not do so more strongly with larger set size, and hence cannot be described as a capacity limit.”

Although Oberauer’s (2019) modeling of the data favored no capacity limit for words, an examination of the evidence made me wonder whether the model simply was unable to detect item capacity limits that might have had subtle effects. For example, Oberauer’s ***Figure 3*** (bottom panel) shows that the proportion of response choices of words not found anywhere in the list increased with list length. Although this proportion was exceptionally low for a large set of words (Experiment 1, left), it rose to almost 10% of responses in some conditions for a small set of words that were often reused from trial to trial (Experiment 2, right). That kind of result, along with past evidence for item capacity limits (such as many of the studies reviewed by Cowan, 2001 and Oberauer et al., 2018), motivated me to search Oberauer’s posted data set more carefully for signs of item limits in his Experiment 2.

## Re-Evaluating Oberauer’s (2019) Findings

Oberauer’s (2019) conclusions present a challenge because it is difficult to understand why memory for items would be limited, but in a manner that that does not change with list length. One way it could occur would be if there were an imperfect, episodic long-term record of a trial that included many, but not all, of its items, e.g., perhaps a proportion of the presented items that is constant across list lengths. This long-term episodic information would be supplemented by serial position information held in a more capacity-limited manner (perhaps with a fixed number of item-to-position bindings for each list, regardless of the list length; or perhaps with a number of bindings that is limited by cross-item interference).

Before accepting Oberauer’s (2019) theoretical solution, however, we need to look carefully for possible subtle evidence of capacity limits for words within a list, in addition to the clear evidence of a capacity limit in the item-to-position binding (which is in no way questioned here). I do this for Oberauer’s Experiment 2, in which it is necessary to have item-to-trial binding to remember items, because of a small pool of items reused from one trial to the next. Item-to-trial binding in this situation is known to be much more difficult than simply remembering which words occurred in the experiment, when the words are drawn from a large pool and words seldom (or never) are repeated from trial to trial, as in Oberauer’s Experiment 1 (e.g., Endress & Potter, 2014; Wolfe, 2012). In Oberauer’s Experiment 2, I re-examine in turn the evidence of capacity limits, the selection of non-list items, and the overall modeling result.

### Assessment of Observed Capacity Limits

**Figure 2:**
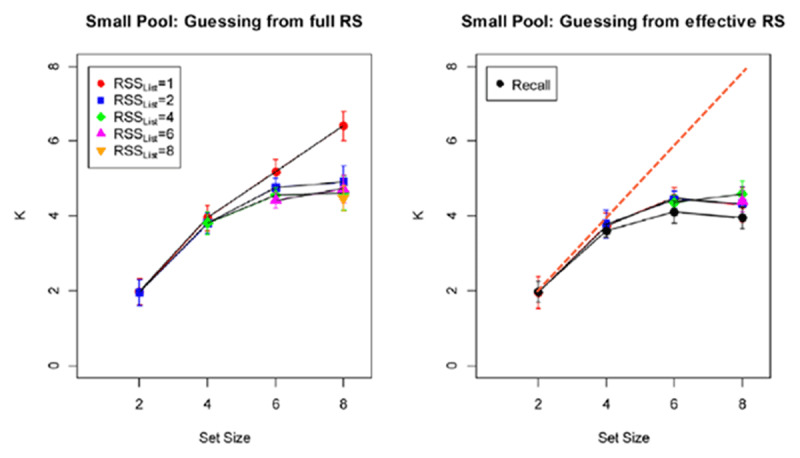
Capacity estimates for guessing from the full response set (left-hand panel) or the effective response set, i.e., excluding lures provided outside of the list items (right-hand panel). Reproduced from Oberauer (2019), Figure 7, bottom half of figure, except that the dashed line on the right has been added to represent the prediction of ceiling-level performance for the RSS_List_ = 1 condition.

Oberauer (2019, Figure 7 and accompanying text, reproduced as the present ***Figure 2***) calculated the number of items in working memory in two ways. The first way (left-hand panel) was under the assumption that guessing took place among all response choices, and the second way (right-hand panel) was under the assumption that guessing took place among the response choices that came from the list. The premise was that the functions should fall on top of one another for the correct capacity metric. The two panels look very similar in most cases. However, there is a large deviation for the case in which there was 1 response choice from the list and 1 response choice that was not in the list, which resulted in excellent performance across list lengths.

For the condition with 1 list item and 1 non-list item as the response choices, the observed capacity in the left-hand panel would be based on a guessing rate of ½. Presumably, the same condition was not plotted in the right-hand panel because it would involve a guessing rate of 1/1, leading to a perfect ceiling effect across all list lengths. But if that ceiling-effect line were included, as shown in the present ***Figure 2***, it would challenge the notion of better convergence with the calculations based on binding.

The reason why excellent performance occurred in this key condition with only one choice from the list and one choice not from the list, much better than in the other conditions of the experiment at longer list lengths, is not clear. Presumably, performance in that condition can be based on item information, and a participant simply determined which word was in the list without needing serial position information. Nevertheless, capacity for items still seems to be limited in this condition.

We can estimate the number of items in working memory *k* at each list length by assuming that correct responding occurred either when the correct item was known, with probability *k*/(list length), or when the correct item was not known but a correct guess was made, with probability [1-*k*/(list length)](.5). These simple assumptions lead to estimates of *k* for list lengths 2, 4, 6, and 8 (with SEM) of 1.97 (.02), 3.95 (.04), 5.19 (.17), and 6.40 (.28), respectively. The flattening of the function at larger list lengths suggests that there is a capacity between 6 and 8 items in this procedure, perhaps similar to Miller’s (1956) capacity of about 7 items, which Cowan (2001) saw as the practical limit when mnemonic strategies were possible.

### Assessment of Effects of Response Choices Outside of the List

One kind of evidence presented by Oberauer (2019) in his Figure 4 and accompanying analyses was that the proportion correct did not change much when more response choices were added that were not in the list. Moreover, any effect of this number of response choices from outside of the list was unchanged across list lengths.

A closer look at the data, however, suggests that when participants did not know the correct answer, the guessing process was often something other than choosing among known list items. The data suggest that many incorrect guesses were carried out among all of the choices, as Oberauer noted. But does this selection of guesses that were not in the list change with list length? I will discuss some evidence that it does, contrary to Oberauer’s claim, after examining the theoretical expectations based on item capacity limits.

#### Theoretical expectations for extra-list responses

In Experiment 2, in which a small pool of words was used and reused among trials, these extra-list choices are plentiful enough to be analyzed, and they presumably reflect uncertainty about whether a particular response choice was presented in the current trial or only in a previous trial, if at all. Information about a capacity limit in items would come from a selection of non-list items among the response choices more often as a function of increasing list lengths (i.e., set sizes), and selection only from list items less often as a function of increasing list lengths because a higher proportion of the list items are forgotten at longer list lengths.

An assumption one might make is that a list item should be preferred to a non-list item whenever it is recognized as such; a recognized word coming from an unknown serial position should be judged to be more likely to have occurred at the probed position than a word that is not recognized at all. If there were a capacity limit on items in Oberauer’s (2019) Experiment 2, it could be considered along with this assumption about responding, to generate expectations. Note that when there are a lot of response choices from the list, it becomes quite likely that at least one of them should be known, which should drastically reduce the selection of non-list items as response choices.

The expectations suggested above heavily depend on the assumption that if at least one list item is recognized, it is selected in preference to any non-list item. This, however, is a dubious assumption to make, for two reasons. First, there may be a process of elimination that can be used. For example, suppose that there are four response choices from the list but the participant does not remember the one that was the correct choice. Among the other three items from the list, suppose that they all are remembered along with the corresponding serial position information, or perhaps approximate serial position information if there are perturbations of serial position in memory (e.g., Lee & Estes, 1981). If the correct item is not remembered, and if all of the other list items among the choices convey serial position information indicating that they are not the correct item matching the probe position, then the participant might select a non-list choice over a choice from the list because there is no *viable* choice from the list. This process is an instance of recall-to-reject (Rotello et al., 2000) for some list items based on their serial position information.

Second, participants might use a decision criterion in which remembered list items are not always preferred over other items. For any remembered item, there is a probability that it might come from the wrong serial position. Participants might use a decision criterion such that a remembered item is more likely to be selected if several of the choices are remembered words, with one of them seeming like a better choice than others.

In the following re-analysis of Oberauer (2019, Experiment 2) I examine a theoretical parameter *pL*. It is an estimate of the proportion of error trials in which a selection was made from among the incorrect list items only, rather than from among all incorrect choices. The purpose of this parameter is to take into account the point that not every selection of an item from the list implies that the list item was known; it could have been selected as a random guess from among all choices. The estimate of *pL* reflects memory for the items and should decrease with increasing list length because more items are forgotten.

To anticipate the way that *pL* will be used, Oberauer’s (2019) data shows a trend in favor of an item capacity limit, but it may be statistically obscured by the uniform assumptions of the overall model. Here, that trend is targeted more specifically and is shown to be reliable. Especially, it shows up more clearly in the error pattern analyzed here than it did in Oberauer’s overall model. One reason the evidence is not presented here within an overall multinomial processing tree model is that there are intricacies in the responding that were not in Oberauer’s model and would overly complexify the model if they were included. In particular, some individuals selected list items as the incorrect answers even less often than would be expected by chance. To explain how this can occur, a recall-to-reject process will be suggested. For example, if an item is known to have occurred in Position 4, then it cannot be the correct response to a probe in Position 2. The present suggestions are overall theoretically friendly to Oberauer’s approach, but with new evidence that the item-to-trial binding limit (aka an item capacity limit) must be taken seriously, not only the item-to-position binding capacity limit that Oberauer established. The contribution is one of revealing an effect that must be considered in future modeling, with some apparent discrepancies between conditions that remain to be explained.

#### Re-analysis of results

In case something like the aforementioned recall-to-reject process is taking place, in my re-analysis of the evidence I minimize the effect of recall-to-reject by excluding individual participants’ estimates for conditions in which the selection of incorrect choices from the list occurred even less frequently than would be expected if participants guessed equally among all choices. When that occurs, it results in a negative *pL* estimate, which was excluded from further comparisons.

Suppose that in a particular condition, a participant’s errors (1-*pCorr*) include some from the list (*pEFL*, or errors from list). We need to estimate a proportion *pL* of error trials in which an erroneous decision is made from the list items rather than from all items. A capacity limit for items implies that, with the number of response choices held constant, *pL* should decrease with list length because of the decreasing chance of knowing list items within the response set. If there were no item capacity limit, *pL* would not decrease with list length when response choices are held constant.

We can first estimate the probability that a guess from all items would result in an error that is a list item. The number of choices includes some list items (*#L*), one of which is the correct answer, and some items that were not on the list (*#N*). An error that is a list item can be made through the process of guessing randomly (*pLRG*, or erroneous list responses from random guesses) such that

1 pLRG = \frac{{\left({\# L - 1} \right)}}{{\left({\# L + \# N - 1} \right)}}

For example, if there are 4 response choices from the list (3 of them incorrect) and 4 response choices not in the list (all of them incorrect) then a random guess will result in an incorrect list item on 3/7 of the error trials. The errors in which a list item was chosen can come from two types of processes, which can be summed:

2 pEFL = pL + \left({1 - pL} \right)\;pLRG

This equation states that when an error is from the list, it could occur because guessing was just from the list, with probability *pL*, or because guessing was from all items, which occurs with probability (1-*pL*) and produces an erroneous list item on *pLRG* of all errors from this guessing method. This equation can be solved for *pL*, the proportion of erroneous guesses made by guessing only among list items:

3 pL = \frac{{pEFL - pLRG}}{{1 - pLRG}}

***Table 2*** shows that *pL* was much lower than would be obtained if participants generally selected from among list items only. Oberauer (2019) also noted this, though the point is more precisely quantified here. Further analyses of *pL* below indicate a potential capacity limit for items.

**Table 2:** Data For lists of 4, 6, or 8 Items Tabulated from Oberauer (2019, Experiment 2), and the Distribution of Response Choices When Both Kinds of Errors were Possible.


List	Response Choices		*pCorr*	p(Errors from List)			n of *pL*	SEM of *pL*
	
Length	In List	Not inList		*pEFL*	*pLRG*	*pL*		

4	2	2	0.95	.02	0.33	0.36 (.68)	10 (7)	0.20 (.16)

4	4	4	0.96	.04	0.43	0.91 (.91)	8 (8)	0.06 (.06)

6	2	2	0.86	.08	0.33	0.48 (.62)	18 (15)	0.11 (.10)

6	4	4	0.80	.14	0.43	0.55 (.59)	17 (16)	0.09 (.08)

6	6	4	0.75	.21	0.48	0.48 (.71)	20 (16)	0.13 (.08)

8	2	2	0.75	.13	0.33	0.20 (.30)	20 (17)	0.07 (.05)

8	4	4	0.62	,24	0.43	0.40 (.48)	20 (18)	0.09 (.08)

8	6	4	0.62	.30	0.56	0.51 (.61)	20 (17)	0.08 (.06)

8	8	4	0.62	.29	0.64	0.40 (.60)	20 (15)	0.10 (.08)


*Note*: Abbreviations: *pCorr* is proportion correct, *pEFL* refers to the proportion of responses that were selections of lures coming from the list, and *pLRG* is the proportion of errors that should be to lures coming from the list if random guessing always occurs among all choices, not just the choices from the list. The term *pL* is the estimated proportion of erroneous responses that occurred through the process of guessing only from the list items, as opposed to guessing from all choices. The number of participants n for *pL* in some conditions is limited because of the exclusion of participants with error-free performance. The numbers in parentheses occur when individual negative estimates of *pL* are omitted. SEM is the standard error of the mean of *pL*. A mixture of trials with guessing from the list items and from all items can be inferred because *pLRG* < *pL* < 1.

Before checking for a capacity limit, individual negative estimates of *pL* were omitted. These negative estimates emerge when the probability of errors coming from the list, *pEFL*, was even smaller than the probability expected if participants always guessed from all response choices when the answer was unknown, *pLRG*. One way this can happen is the aforementioned strategy in which known list items thought to have come from serial positions other than the probed position can be ruled out by a recall-to-reject process (Rotello et al., 2000). The effects of negative estimates are indicated in ***Table 2*** by the lower participant numbers in parentheses when these negative estimates are excluded.

**Figure 3:**
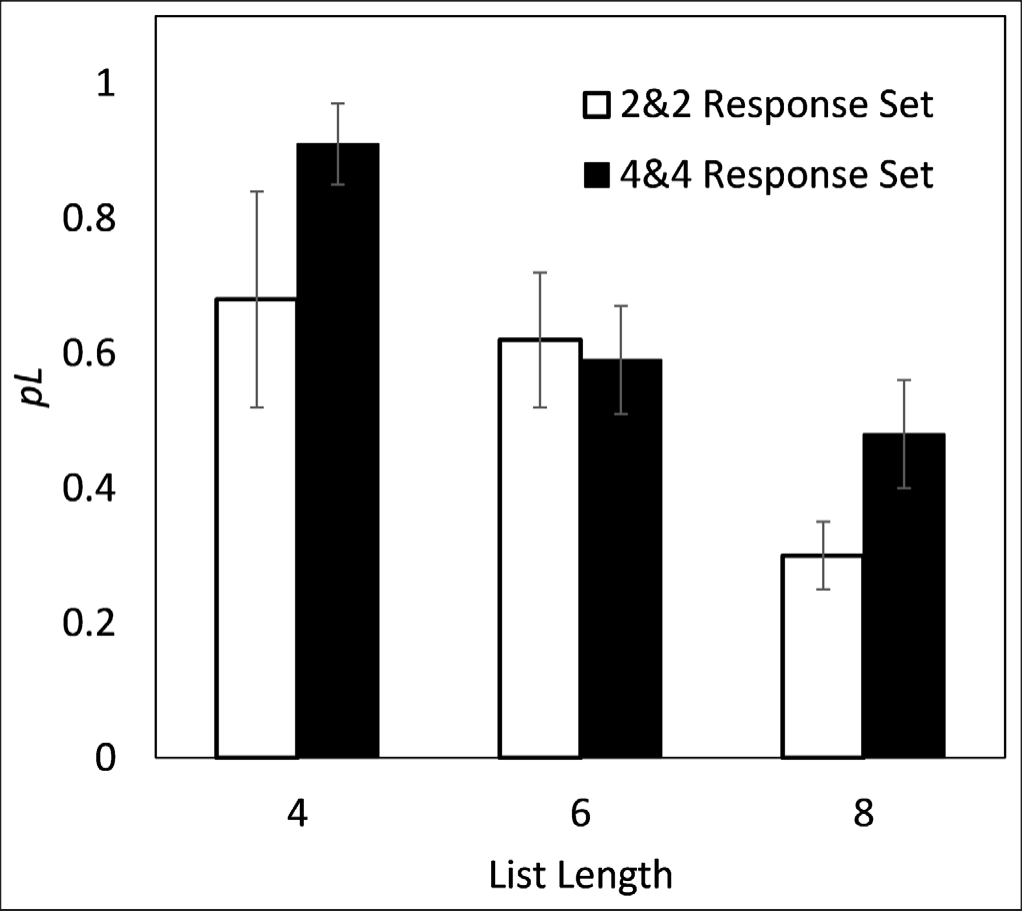
The decrease in the mean *pL*, the estimated proportion of erroneous responses based on guessing from only the list items, as a function of list length with two different response sets (trials with 2 items from the list and 2 not from the list, and trials with 4 items from the list and 4 not from the list). The graph is based on the data depicted in parentheses in Table 2, with estimates of *pL* below zero removed. Error bars reflect plus and minus one standard error of the mean.

The data can be used to re-evaluate the statement of Oberauer (2019, p. 10) that “whatever limits item memory does not do so more strongly with larger set size, and hence cannot be described as a capacity limit.” Contrary to this statement, the value of *pL* generally did grow smaller as the list length grew from 4 to 6 to 8 (***Figure 3***). A slope across list lengths was calculated; negative values of *pL* were not used to calculate this slope because they indicate a likely recall-to-reject strategy.

Analyses were conducted with a Cauchy prior (scale 0.707) using JASP Version 0.14.1 (JASP Team, 2020). This slope across list lengths was negative for trials with 2 choices within the list and 2 choices outside of the list, M = –.16, t(16) = 3.31, BF = 10.57, reliably below zero. ***Table 2*** shows that with 4, 6, and 8 list items, respectively, the relevant *pL* = .68, .62, and .30. The slope was also negative with 4 choices within the list and 4 choices outside of the list, M = –.12, t(16) = 2.72, BF = 3.81. As shown in the table, for 4, 6, and 8 list items, respectively, *pL* = .91, .59, and .48. There was no reliable slope across list lengths for trials with 6 items from within the list and 4 items from outside of the list, M = –.02, BF = 0.29. With those response choices, for List Lengths 6 and 8 , *pL* = .71 and .61, respectively. However, averaging each participant’s slope across the three controlled response display situations, including every condition in which the participant had a non-negative value of *pL*, the average slope M = –.11 (95% credible interval –.17 to –.04) was reliably below zero, t(18) = 3.57, BF = 18.78. This slope was below zero also according to a nonparametric test, namely a sign test (1-tailed, p < .025), with negative slopes in 13 participants, positive slopes in 4, and slopes of 0.00 that were not counted one way or the other in the remaining 2 participants.

The magnitude of the slopes is not completely consistent with a model in which participants guess only from list items when at least one list item is remembered. The proportion of error trials in which no list item was recognized is (1-*k*/*L*)*^R^*, where *k* is the number of items in working memory (not necessarily including the serial position information), *L* is the list length, and *R* is the number of incorrect choices that come from the list. This formula follows from the point that *k* of *L* list items are held in working memory (by the definition of *k*) so that for any item, the probability that it is not held in working memory is 1-*k*/*L*. Among *R* incorrect response choices drawn from the list, the probability that none of them will be remembered is (1-*k*/*L*)*^R^*. Accordingly, the proportion of error trials in which at least one incorrect list item was recognized as being from the list would equal one minus this quantity, or 1-(1-*k*/*L*)*^R^*. This is an estimate of *pL* under Oberauer’s assumption in his model that, if at least one item from the list is recognized, one of them will be selected over all of the response choices that were not drawn from the list, even when the item coming from the correct serial position is not remembered. For example, suppose *k* = 4 (Cowan, 2001). For two choices from the list (one incorrect) and two not from the list, the predicted slope as *L* increases from 4 to 6 to 8 is –.13, in fairly good accord with the results (–.16; 95% credible interval –.26 to –.06). However, for four choices from the list (three incorrect, so *R* = 3) and four not from the list, the predicted slope is only –.03, at the low boundary of the credible interval for the actual obtained value (–.12; 95% credible interval –.22 to –.03). For six choices from the list (five incorrect) and four not from the list, the predicted slope is only –.01, which is consistent with the results (–.02; 95% credible interval –.15 to +.10). Models with other values of *k* produce flatter slopes, i.e., less change across list lengths than we observed, and their agreement with the actual results is therefore worse. The discrepancy between the data and expectations for four choices from the list and four not from the list, with a greater effect of list length than expected, suggests that participants may have used a decision criterion in which responding with a list item was more likely when more than one list item was remembered.

It is not clear why the indirectly inferred capacity based on these *pL* estimates (4) is so much lower than than the estimated capacity when there is only one choice from the list and one choice from outside the list and the capacity was directly estimated (6.4 at List Length 8, in the previous section). It may be that the need to evaluate multiple response choices causes forgetting of some items (cf. Luck & Vogel, 1997).

We might rely on Oberauer’s (2019) serial recall data to indicate what is known, free of cues or interference from recognition choices. In the serial recall condition, the mean error proportions for List Length 4 were .04 from within the list and .03 from outside of the list; for List Length 6, they were .15 and .11; whereas for List Length 8, they were .32 and .13. The increase in the proportion of words recalled from outside of the list when the list length increased from four items (.03) to six and eight items (.11 and .13, respectively) might occur because processing longer lists brings in executive processes that compete with word encoding at some specific transition point during the list presentation, when a simple capacity is exceeded and a more demanding form of maintenance first comes into play, presumably after 3 to 5 words (Barrouillet et al., 2021; Cowan, 2001; Vergauwe et al., 2014).

### Assessment of Models of Performance

In a multinomial processing tree model of recognition presented by Oberauer (2019), knowledge of the binding of the correct word to its serial position was said to lead to the correct response. In the absence of that knowledge, memory of the correct word without its binding information was said to narrow down the choices to items that were in the list, making a correct guess more likely than would be a random guess among all choices. In the absence of item knowledge, a random guess among all choices takes place. Based on the model, both item and binding information may have changed with set size (list length) in this experiment, though more severely for binding information.

The change in item memory across list lengths did not seem statistically reliable in Oberauer’s model but still might reflect an important capacity limit to be established. Oberauer’s Figure 5 shows that, in Experiment 2, the likelihood of knowing an item decreased from about .62 for 2-item lists to about .45 for 8-item lists. This corresponds to a loss of 1.36 of the items from an 8-item list due to the list length [i.e., (.62–.45)8 = 1.36]. Adjusting the performance levels to compensate for the non-ceiling level of performance even at the shortest list length, this would place capacity at 8–1.36 = 6.64 items, an estimate similar to the 6.4 items suggested above on the basis of 2-choice data.

## Concluding Comments

In the study of Oberauer et al. (2019, Experiment 2), it was found that response choices that were not in the list did not influence accuracy in a manner that depended on the list length. Here I show that these response choices from outside of the list did influence the distribution of error responses, with proportionally more guessing from outside of the list at longer list lengths in some response set conditions. Some estimates of item capacity from the data suggest that between 6 and 7 items were remembered. It is not fully clear whether the item capacity limit in some other circumstances is actually more severe than that, or whether further modeling refinement would allow the estimates to converge.

There is still more that needs to be understood about why items from the list were not chosen more consistently among wrong answers. One possibility that does not appear to be represented in the multinomial processing tree model that Oberauer (2019) presented is this: A participant might know that some words from the list come from the wrong serial positions and therefore can be ruled out as response choices. It is tricky to model this kind of process of elimination, a recall-to-reject process (Rotello et al., 2000), inasmuch as the use of such indirect information may be intermittent. There have, though, been other successful models incorporating the process of elimination and informed guessing (e.g., Cowan et al., 2013, 2016; Rhodes et al., 2018).

The finding that word knowledge appears either limited or unlimited, depending on the circumstances (in Oberauer 2019, Experiment 1 versus Experiment 2), was seen also by Cowan et al. (2012). In that study, lists of words and/or integrated multiword chunks (e.g., *leather brief case*) were followed by a test in which a word from the list was paired with a word from outside of the list, the task being to select the word that was from the list, with no requirement to access the serial position information. In most experimental conditions, the model suggested that participants retained only about 3 chunks regardless of chunk length. However, long lists of singletons showed much better performance than expected, similar to the present condition with 1 response choice from the list (i.e., the correct answer) and 1 other response choice not from the list. Cowan et al. (2012) concluded that a chunk-capacity-limited mechanism was supplemented by an activated long-term memory component (with rapid learning of new material) that can be used to retain strings of words, and that notion could explain the excellent performance in Oberauer’s (2019) Experiment 1. Perhaps that long-term component involves semantic aspects of the words (e.g., Poirier & Saint-Aubin, 1995).

In sum, a re-analysis of the data from Oberauer (2019) suggests that the notion of a capacity limit for lists based on bindings must include not only item-to-serial-position binding, but also item-to-list binding that amounts to an item capacity limit, when items are reused from trial to trial. The exact limit remains to be identified. It is not clear whether the capacity limit is less severe when there are fewer response choices, and particularly when the correct choice must be discriminated only from a non-list item. In that situation, performance might be helped by a recent-familiarity signal (Mickes et al., 2013; Oberauer, 2018), which would be less effective when some of the incorrect choices are list items. The role of serial position information in eliminating incorrect choices through a recall-to-reject process also should be considered in future work. I hope that making these possibilities more salient will encourage research to answer still-unaddressed, fundamental issues regarding capacity limits for words and their serial position bindings.
